# Supplementary material for: Modeling Continuous Admixture Using Admixture-Induced Linkage Disequilibrium
Source: Sci Rep. 2017 Feb 23;7:43054. doi: 10.1038/srep43054 (PMC5322361; doi:10.1038/srep43054)
Supplement: Supplementary Information [file srep43054-s1.pdf]

Supplementary Information For Dating continuous admixture

Ying Zhou, Hongxiang Qiu, Shuhua Xu

January 14, 2017

List of Tables

S1 Adjusted p-values of pairwise Wilcoxon signed rank test with HI, GA, CGF1 and CGF2 as core models 1
S2 Admixture inference with AdmixInfer. 2

List of Figures

S1 Evaluation of CAMer under various simulated admixture models, core models are HI, GA, CGF1 and CGF2. 3
S2 Admixture inference on simulation data under HI (100) model. 4
S3 Admixture inference on simulation data under HI (50) model. 5
S4 Admixture inference on simulation data under CGF1 (1-100) model. 6
S5 Admixture inference on simulation data under CGF1 (1-50) model. 7
S6 Admixture inference on simulation data under GA (1-100) model. 8
S7 Admixture inference on simulation data under GA (1-50) model. 9
S8 Admixture inference on simulation data under CGF1-I (30-100) model. 10
S9 Admixture inference on simulation data under CGF1-I (70-100) model. 11
S10 Admixture inference on simulation data under GA-I (30-100) model. 12
S11 Admixture inference on simulation data under GA-I (70-100) model. 13
S12 Admixture inference on the population ASW from HapMap. . 14
S13 Admixture inference on the population ASW from 1KG. . . . 15
S14 Admixture inference on the population MEX from HapMap. . 16
S15 Admixture inference on the population Hazara from HGDP. . 17
S16 Admixture inference on the population Uyghur from HGDP. . 18
S17 Admixture inference on the population MKK from HapMap. . 19

Table S1: Adjusted p-values of pairwise Wilcoxon signed rank test with HI, GA, CGF1 and CGF2 as core models

True model	Best models	Adjusted p-Values of Pairwise Wilcoxon Signed Rank Test					
		HI:GA	HI:CGF1	HI:CGF2	GA:CGF1	GA:CGF2	CGF2:CGF1
HI (100)	HI	0.012	0.012	0.012	0.012	0.012	0.012
HI (50)	HI	0.012	0.012	0.012	0.012	0.012	0.012
CGF1 (1-100)	CGF1, CGF2	0.020	0.012	0.012	0.012	0.012	0.064
CGF1 (1-50)	CGF1, CGF2	0.039	0.012	0.012	0.018	0.012	0.19
GA (1-100)	GA	0.012	0.012	0.012	0.012	0.012	0.012
GA (1-50)	CGF2, GA	0.012	0.012	0.012	0.39	0.39	0.012
CGF1-I (30-100)	HI	0.012	0.012	0.012	0.012	0.012	0.012
CGF1-I (70-100)	HI	0.012	0.012	0.012	0.012	0.012	0.012
GA-I (30-100)	HI	0.012	0.63	0.039	0.012	0.012	0.012
GA-I (70-100)	HI	0.012	0.012	0.012	0.012	0.012	0.012

Table S2: Admixture inference with AdmixInfer.

True model	True date(s)	Best model	Estimation	
			mean	CI
HI	100	HI	99.55	(99.31, 99.79)
HI	50	HI	50.15	(49.98, 50.32)
CGF1	100	CGF1	96.95	(96.85, 97.05)
CGF1	50	CGF1	49.9	(49.76, 50.04)
GA	100	GA	96.5	(96.26, 96.74)
GA	50	GA	48.7	(48.48, 48.92)
CGF1-I	(30,100)	HI	73	(73, 73)
CGF1-I	(70,100)	HI	88.15	(87.98, 88.32)
GA-I	(30,100)	HI	75	(75, 75)
GA-I	(70,100)	HI	89.2	(89.01, 89.39)

Inference was based on the ancestral segments generated by AdmixSim under all admixture models as we discussed in the main text. The simulated time or time interval is in the column of "true date(s)", the estimated means and 95% CIs for the admixture starting time are listed in the last two columns. All of these time estimations are measured by generation.

Figure S1: Evaluation of CAMer under various simulated admixture models, core models are HI, GA, CGF1 and CGF2.

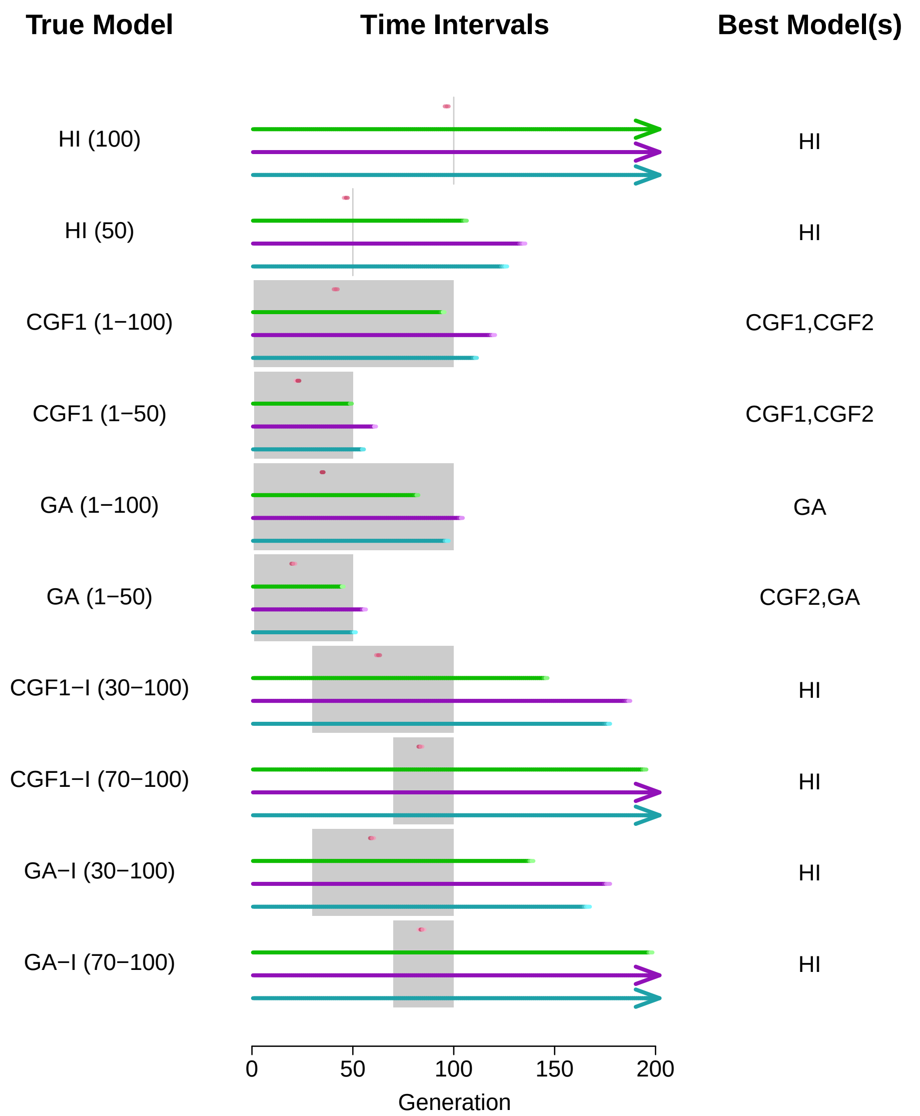

The simulated models (True Model) coded as in Simulation studies section are listed on the left, with the admixture time intervals in the parentheses. Gray areas in the middle vertical panel are the simulated time intervals, while colored lines are the estimated time intervals under different core models. HI: pink; CGF1: green; CGF2: purple; GA: blue. The intensity of lines means the number each point is covered by the time intervals estimated from all jackknives. Lighter colors represent fewer covers while darker colors mean more. And the arrow means the beginning of the admixture older than 200 generations ago.

Figure S2: Admixture inference on simulation data under HI (100) model.

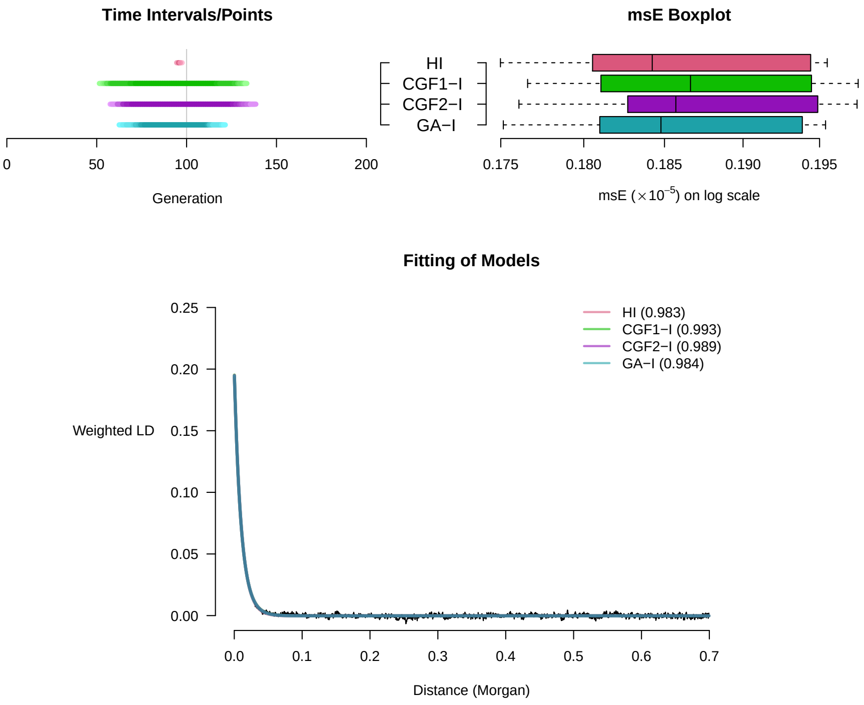

Top left: Time intervals for admixtures are plotted as color horizontal bar. Bottom: Weighted LD (refers to $Z(d)$, in black solid line) is fitted under different models, with F values in the brackets. Core models used for inference: HI, GA-I, CGF1-I, and CGF2-I.

Figure S3: Admixture inference on simulation data under HI (50) model.

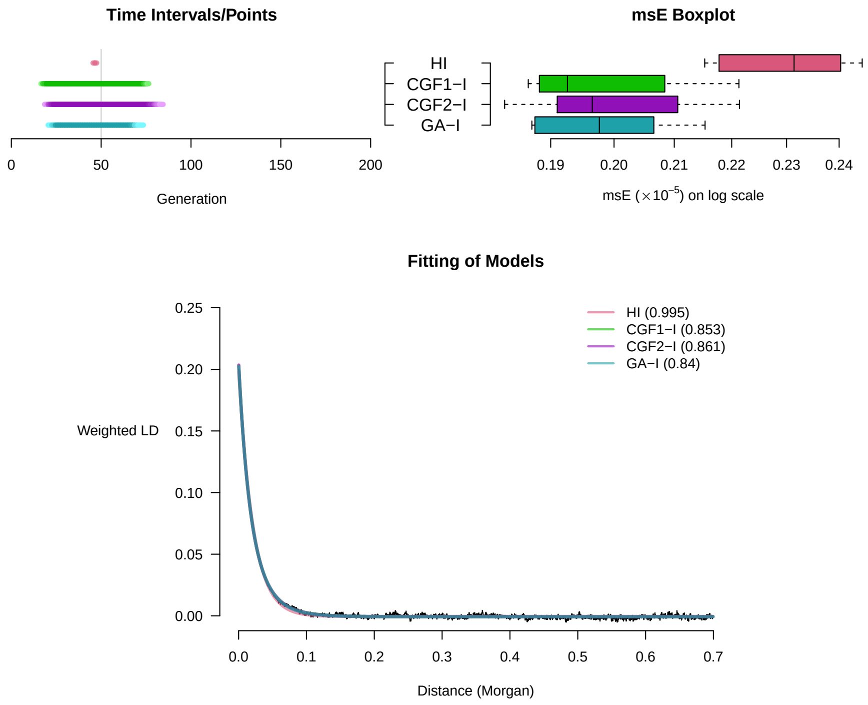

Top left: Time intervals for admixtures are plotted as color horizontal bar. Bottom: Weighted LD (refers to $Z(d)$, in black solid line) is fitted under different models, with F values in the brackets. Core models used for inference: HI, GA-I, CGF1-I, and CGF2-I.

Figure S4: Admixture inference on simulation data under CGF1 (1-100) model.

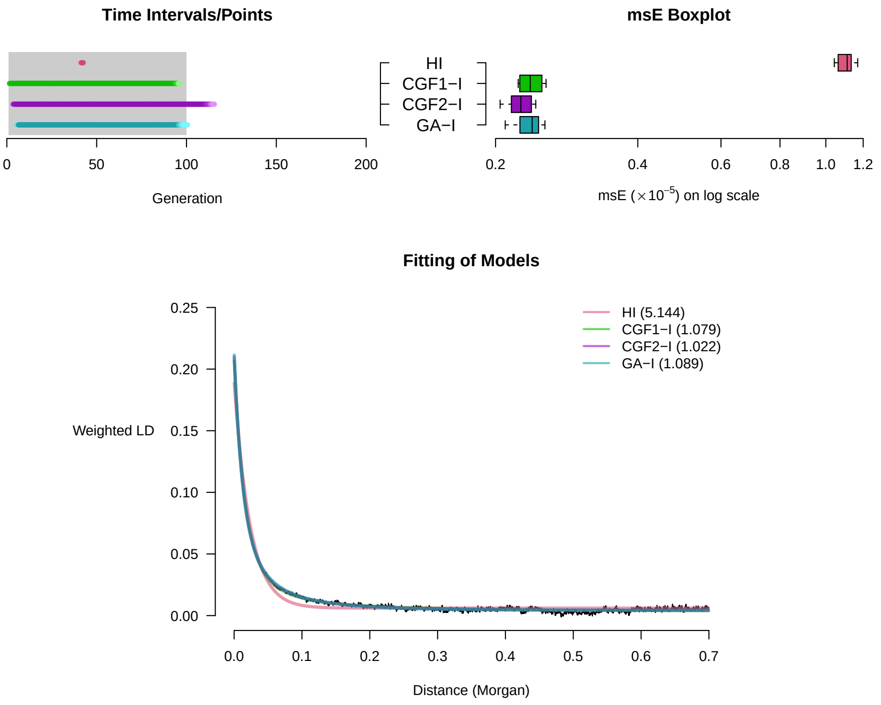

Top left: Time intervals for admixtures are plotted as color horizontal bar. Bottom: Weighted LD (refers to $Z(d)$, in black solid line) is fitted under different models, with F values in the brackets. Core models used for inference: HI, GA-I, CGF1-I, and CGF2-I.

Figure S5: Admixture inference on simulation data under CGF1 (1-50) model.

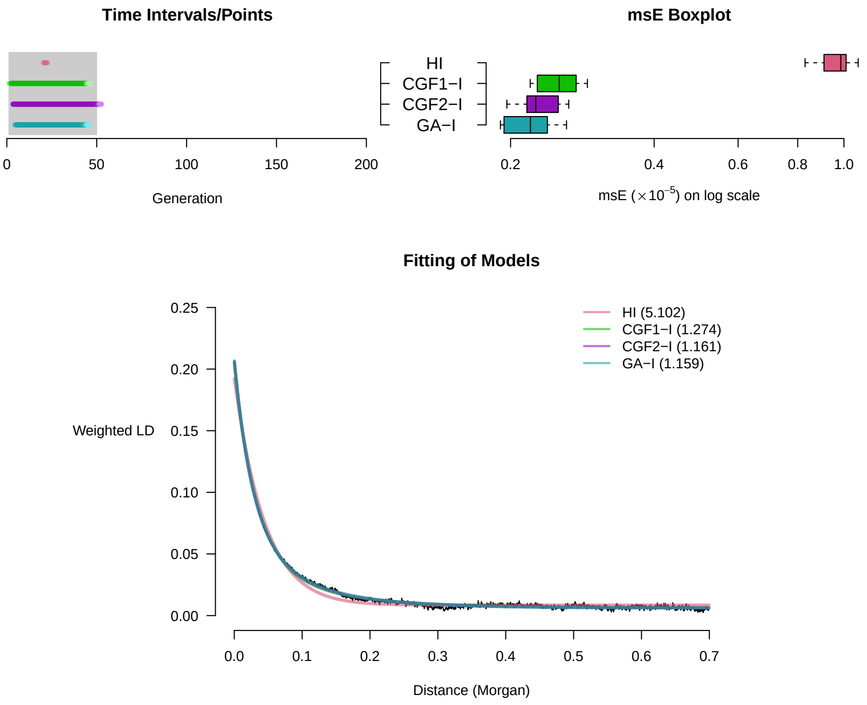

Top left: Time intervals for admixtures are plotted as color horizontal bar. Bottom: Weighted LD (refers to $Z(d)$, in black solid line) is fitted under different models, with F values in the brackets. Core models used for inference: HI, GA-I, CGF1-I, and CGF2-I.

Figure S6: Admixture inference on simulation data under GA (1-100) model.

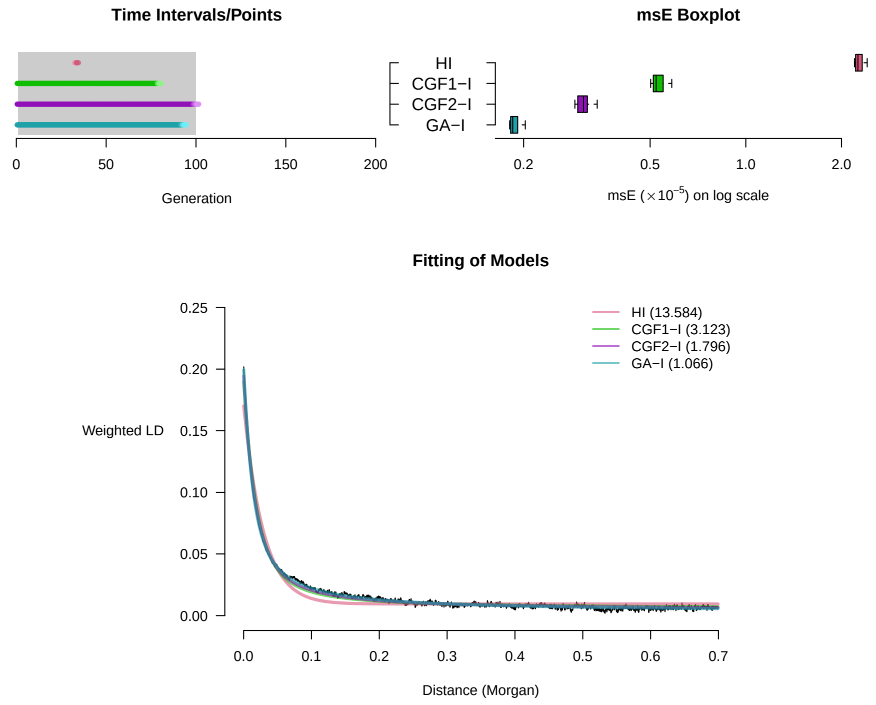

Top left: Time intervals for admixtures are plotted as color horizontal bar. Bottom: Weighted LD (refers to $Z(d)$, in black solid line) is fitted under different models, with F values in the brackets. Core models used for inference: HI, GA-I, CGF1-I, and CGF2-I.

Figure S7: Admixture inference on simulation data under GA (1-50) model.

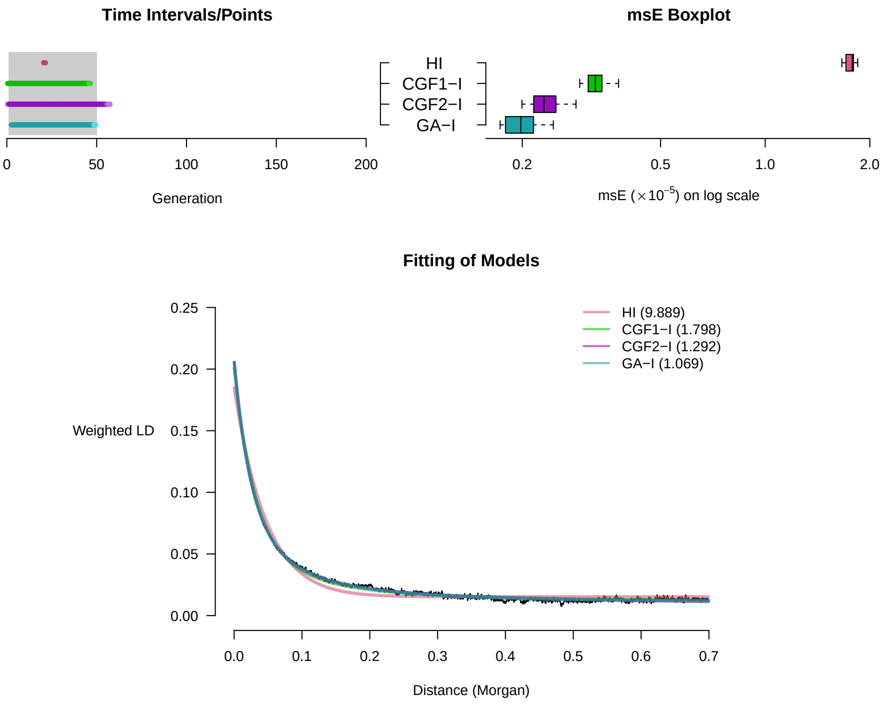

Top left: Time intervals for admixtures are plotted as color horizontal bar. Bottom: Weighted LD (refers to $Z(d)$, in black solid line) is fitted under different models, with F values in the brackets. Core models used for inference: HI, GA-I, CGF1-I, and CGF2-I.

Figure S8: Admixture inference on simulation data under CGF1-I (30-100) model.

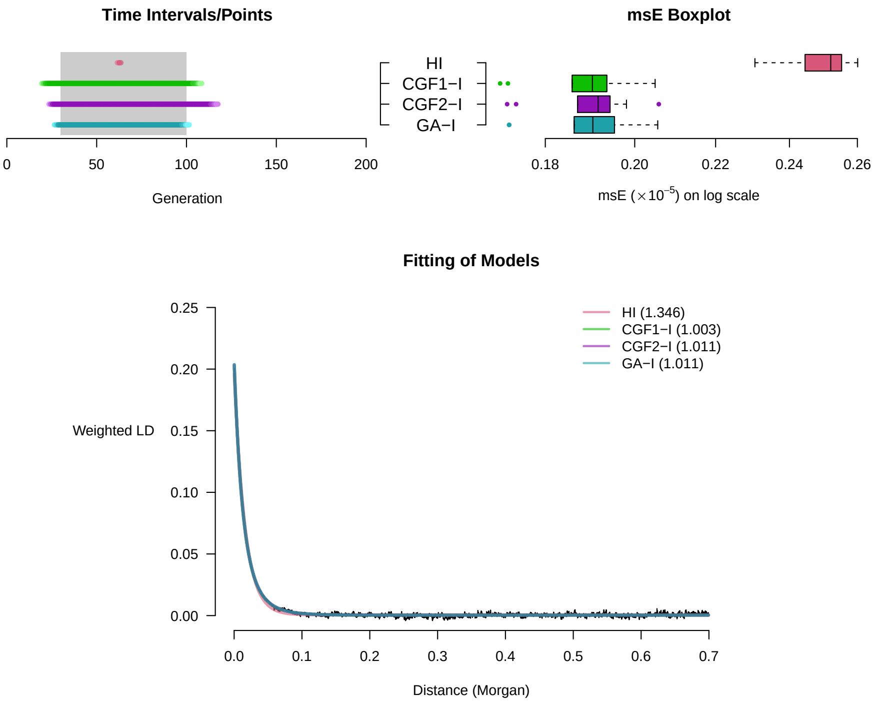

Top left: Time intervals for admixtures are plotted as color horizontal bar. Bottom: Weighted LD (refers to $Z(d)$, in black solid line) is fitted under different models, with F values in the brackets. Core models used for inference: HI, GA-I, CGF1-I, and CGF2-I.

Figure S9: Admixture inference on simulation data under CGF1-I (70-100) model.

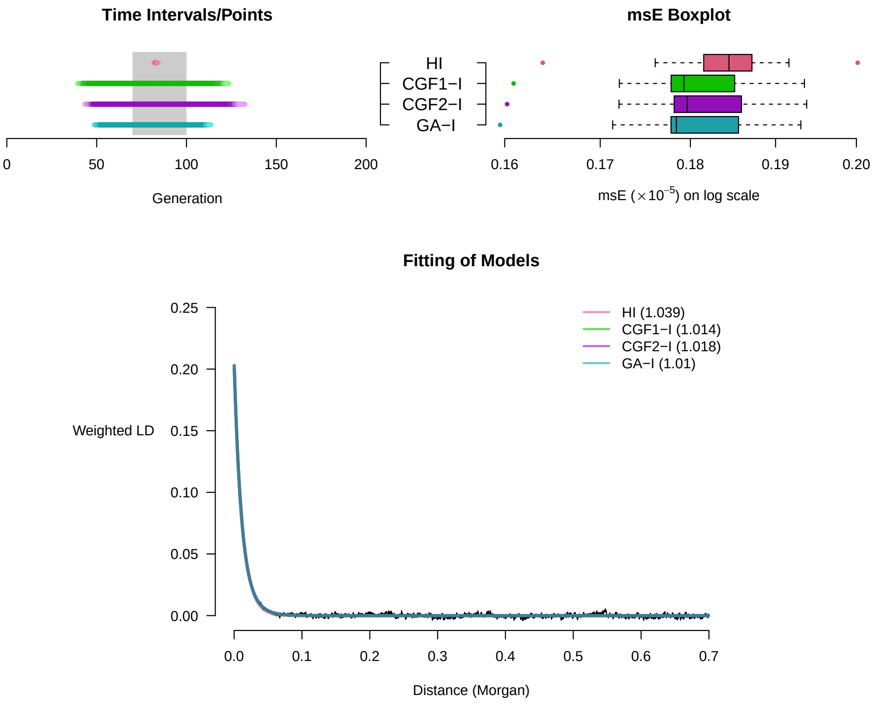

Top left: Time intervals for admixtures are plotted as color horizontal bar. Bottom: Weighted LD (refers to $Z(d)$, in black solid line) is fitted under different models, with F values in the brackets. Core models used for inference: HI, GA-I, CGF1-I, and CGF2-I.

Figure S10: Admixture inference on simulation data under GA-I (30-100) model.

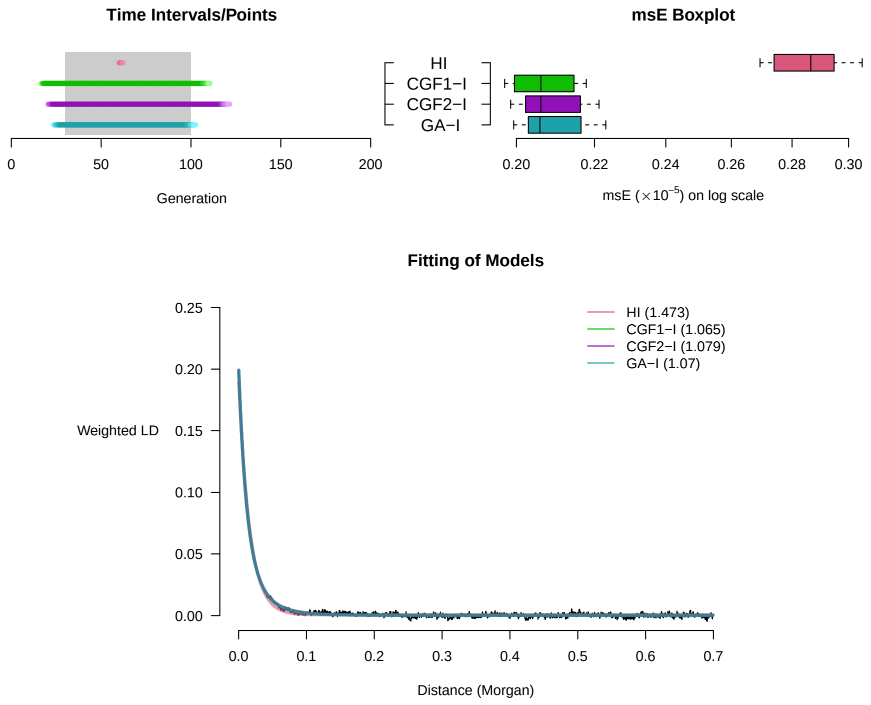

Top left: Time intervals for admixtures are plotted as color horizontal bar. Bottom: Weighted LD (refers to $Z(d)$, in black solid line) is fitted under different models, with F values in the brackets. Core models used for inference: HI, GA-I, CGF1-I, and CGF2-I.

Figure S11: Admixture inference on simulation data under GA-I (70-100) model.

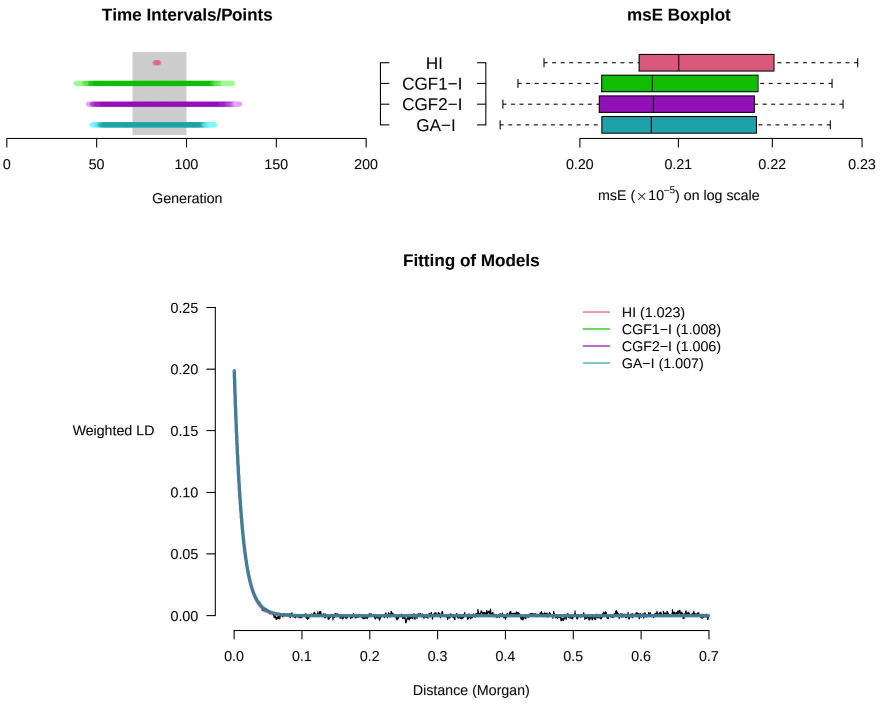

Top left: Time intervals for admixtures are plotted as color horizontal bar. Bottom: Weighted LD (refers to $Z(d)$, in black solid line) is fitted under different models, with F values in the brackets. Core models used for inference: HI, GA-I, CGF1-I, and CGF2-I.

Figure S12: Admixture inference on the population ASW from HapMap.

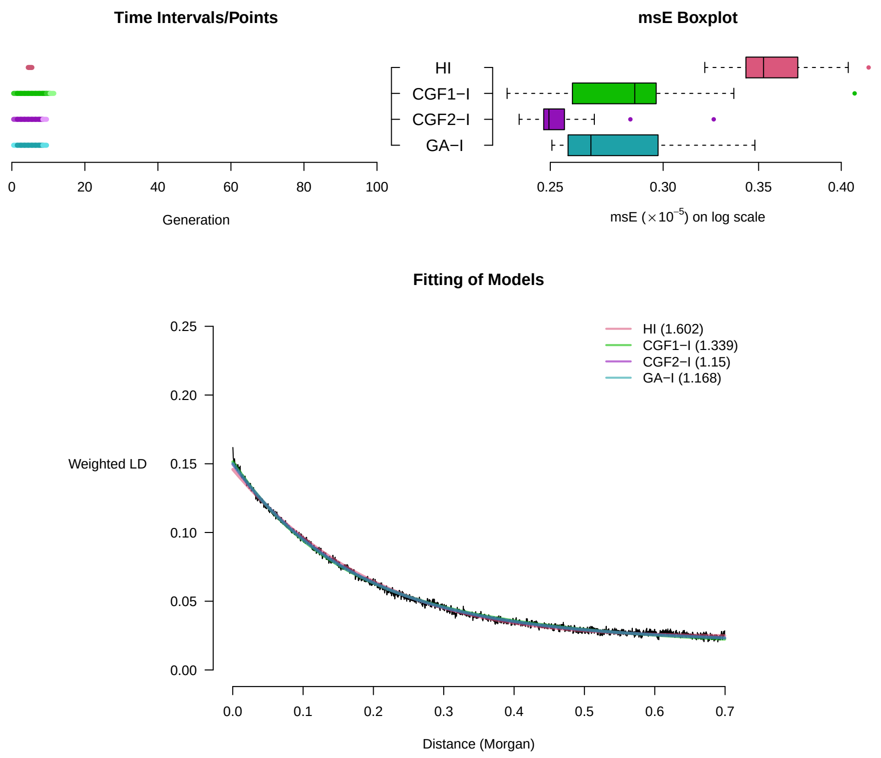

YRI and CEU are usde as reference populations. Top left: Time intervals for admixtures are plotted as color horizontal bar. Bottom: Weighted LD (refers to $Z(d)$, in black solid line) is fitted under different models, with F values in the brackets. Core models used for inference: HI, GA-I, CGF1-I, and CGF2-I.

Figure S13: Admixture inference on the population ASW from 1KG.

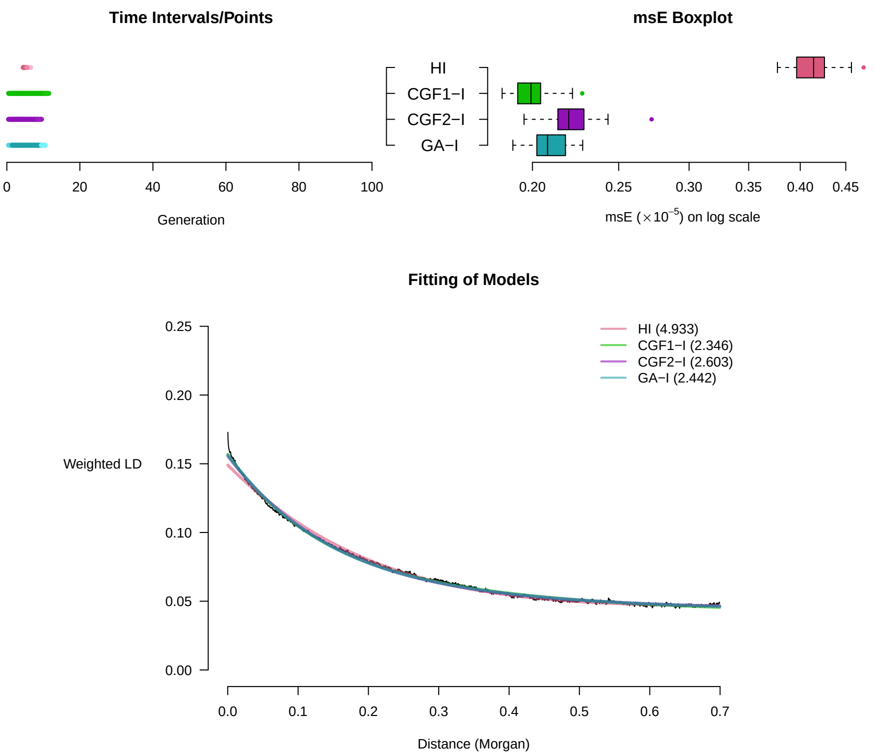

YRI and CEU are usde as reference populations. Top left: Time intervals for admixtures are plotted as color horizontal bar. Bottom: Weighted LD (refers to $Z(d)$, in black solid line) is fitted under different models, with F values in the brackets. Core models used for inference: HI, GA-I, CGF1-I, and CGF2-I.

Figure S14: Admixture inference on the population MEX from HapMap.

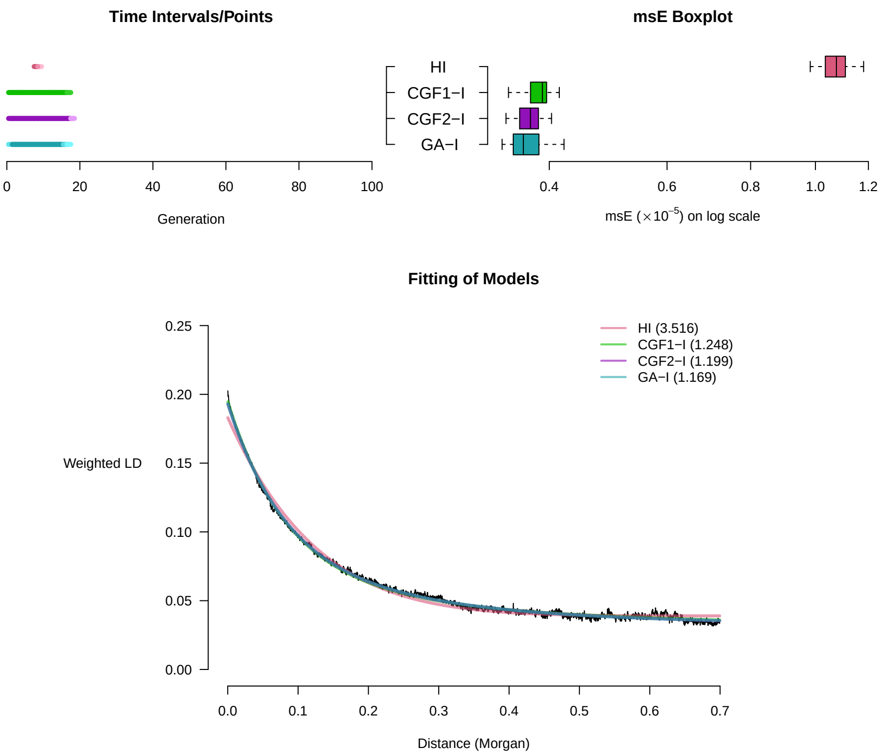

We used CEU to represent European ancestry and combined 7 Colombians, 14 Karitiana, 21 Maya, 14 Pimas and 8 Suruis from HGDP as the American Indian ancestry. Top left: Time intervals for admixtures are plotted as color horizontal bar. Bottom: Weighted LD (refers to $Z(d)$, in black solid line) is fitted under different models, with F values in the brackets. Core models used for inference: HI, GA-I, CGF1-I, and CGF2-I.

Figure S15: Admixture inference on the population Hazara from HGDP.

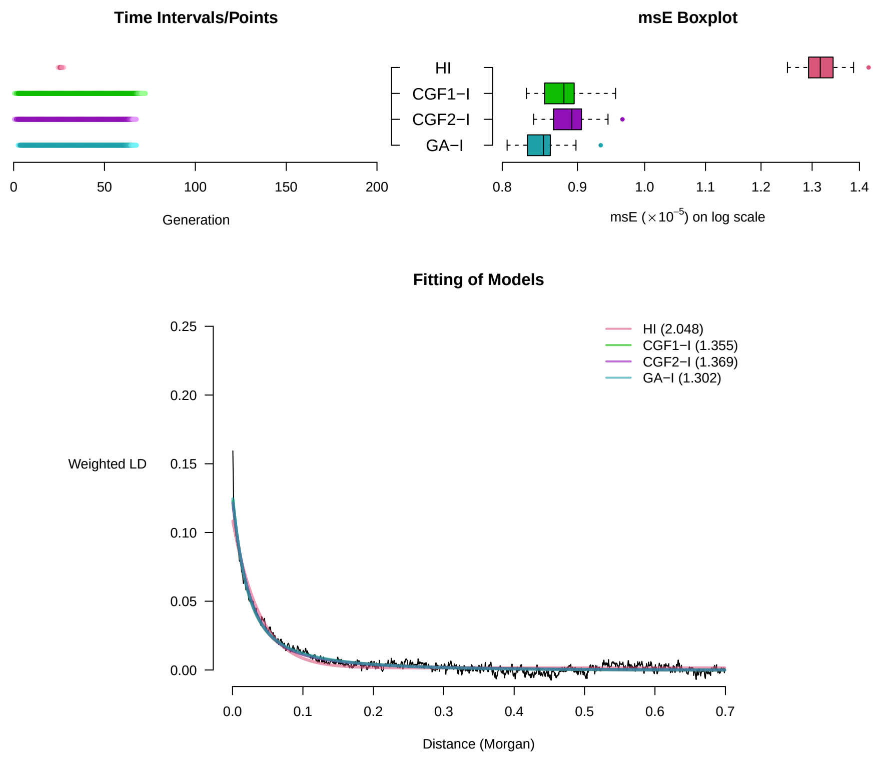

Han and French were used as reference populations. Top left: Time intervals for admixtures are plotted as color horizontal bar. Bottom: Weighted LD (refers to $Z(d)$, in black solid line) is fitted under different models, with F values in the brackets. Core models used for inference: HI, GA-I, CGF1-I, and CGF2-I.

Figure S16: Admixture inference on the population Uyghur from HGDP.

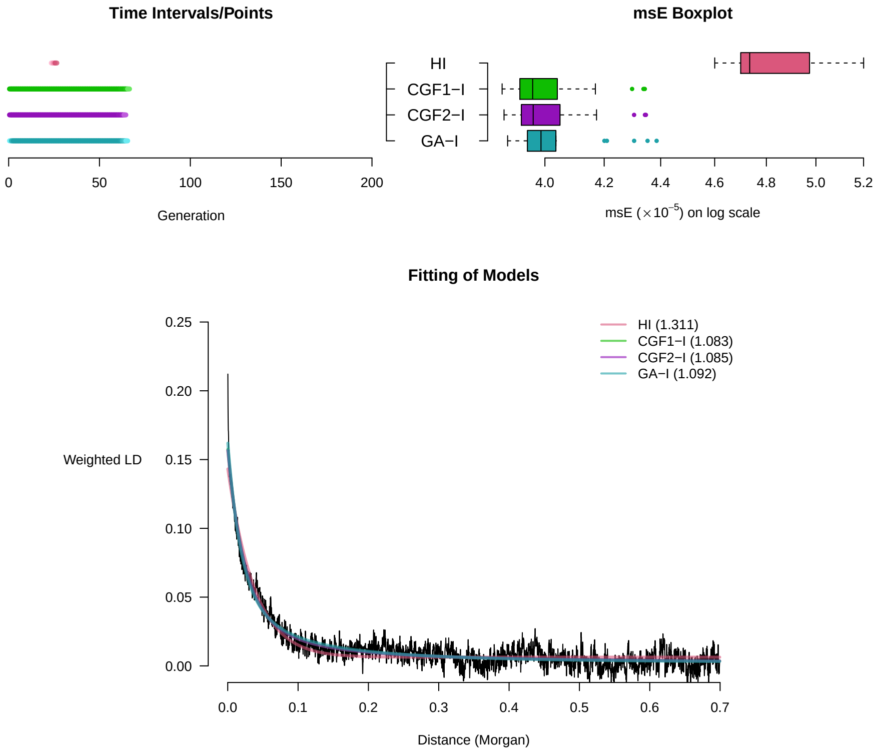

Han and French were used as reference populations. Top left: Time intervals for admixtures are plotted as color horizontal bar. Bottom: Weighted LD (refers to $Z(d)$, in black solid line) is fitted under different models, with F values in the brackets. Core models used for inference: HI, GA-I, CGF1-I, and CGF2-I.

Figure S17: Admixture inference on the population MKK from HapMap.

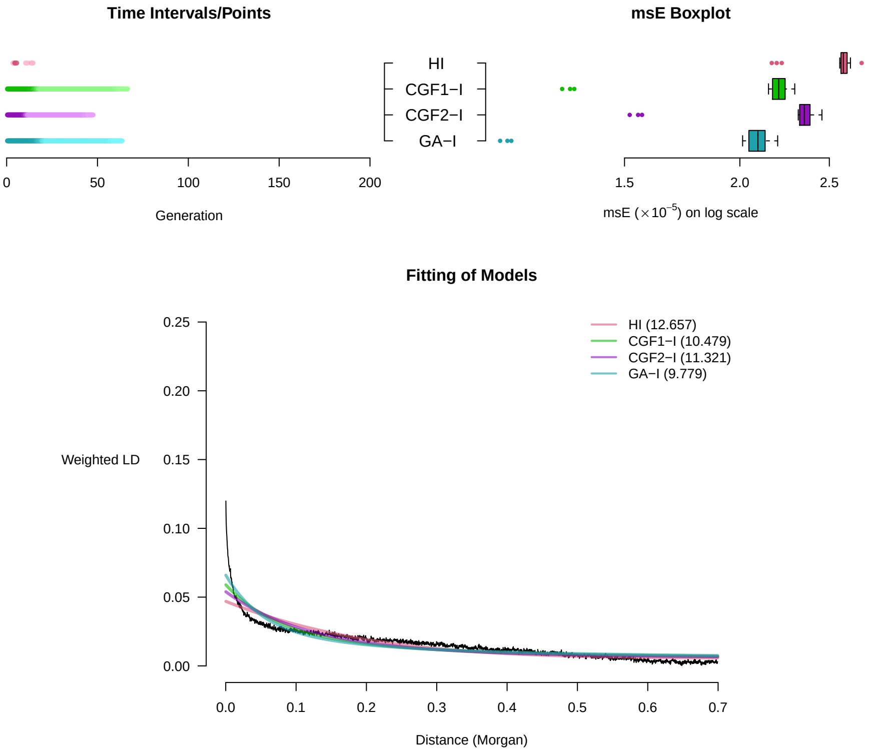

YRI and CEU were used as reference populations. Top left: Time intervals for admixtures are plotted as color horizontal bar. Bottom: Weighted LD (refers to $Z(d)$, in black solid line) is fitted under different models, with F values in the brackets. Core models used for inference: HI, GA-I, CGF1-I, and CGF2-I.
